# Gram stain and addition of amphotericin B to improve the microbial safety of human donor corneas

**DOI:** 10.1007/s10561-021-09981-1

**Published:** 2021-11-17

**Authors:** Davide Camposampiero, Adriano Fasolo, Giuseppe Saccon, Pietro M. Donisi, Elisa Zanetti, Diego Ponzin

**Affiliations:** 1 The Veneto Eye Bank Foundation, Padiglione. G. Rama, Via Paccagnella 11, Zelarino, 30174 Venice, Italy; 2 Pathological Anatomy Unit, SS. Giovanni e Paolo Hospital, Aulss3 Serenissima, Venice, Italy

**Keywords:** Amphotericin B, Donor cornea, Eye banking, Gram stain, Organ culture, Sterility test

## Abstract

To determine the effectiveness of two methods to improve the microbial safety of human corneas preserved in organ culture. We compared the number of positive preservation solutions of corneas in organ culture in which the initial short-term hypothermic corneal maintenance solution was supplemented with amphotericin B 2.5 µg/mL and the historical data of microbial test results (2015–2019). In addition, we appraised the efficacy of Gram stain to detect bacterial or fungal contamination in the organ culture solutions of corneas from at-risk donors compared to the culture tests of corneas from not-at-risk donors. Statistical analysis was performed using STATA and statistical significance set at *p* < 0.05. The number of positive culture tests after preservation was 15 (0.5%) in 2020 compared to a mean of 37 (1.2%) in the period 2015–2019 (*p* < 0.01), with 10 (1.0%) positive samples in the cohort of 998 corneas from at-risk donors and 5 (0.2%) in the 2046 corneas from not-at-risk donors (*p* < 0.01). All corneas from at-risk donors tested positive at Gram stain and the results were available 1–3 days before those of the conventional culture tests. Amphotericin B supplementation in the short-term maintenance solution markedly reduced the number of positive microbial tests after organ culture and the early detection of contaminants, including slow-growing microorganisms, by Gram stain before the standard culture results. This meant fewer corneas being discarded and a greater likelihood of preventing post-graft infections.

## Introduction

Bacterial and fungal contamination of human donor corneas and the risk of post-keratoplasty infections that merit investigation (Kitazawa et al. 2017; Thareja et al. 2020; Thomas 2003). A study comprising 4410 organ-cultured corneas (31 °C) reported an overall microbial contamination rate of 2.5%, with *Candida* spp. accounting for most contaminations (Ling et al. 2019). According to systematic reporting by the Eye Bank Association of America, the yearly incidence of fungal infection after transplantation of hypothermic preserved corneas rose from 0.009% between 2005 and 2007 to 0.016% between 2008 and 2010 (Aldave et al. 2013). Between 2007 and 2014, adverse reactions after corneal transplantation due to fungal infection occurred in half of cases of endophthalmitis- and keratitis-associated pathogens, with no statistical difference between endothelial and penetrating keratoplasty (Edelstein et al. 2016).

Microbial testing of organ-culture preservation solutions is key to detecting and discarding contaminated corneas prior to distribution for transplant, whereas postoperative rim culturing is recommended to warn of the potential risk of infection (Camposampiero et al. 2015; EEBA 2020; Zanetti et al. 2005). Although storage solutions contain empiric broad-spectrum antibiotics, organ culture is still subject to microbial contamination due to media composition and conditions conducive to growth: temperature between 30 and 37 °C and length of preservation time (Thareja et al. 2020). The addition of antibiotics to storage medium is an unreliable remedy due to the increasing antibiotic resistance of contaminating microbes (Li et al. 2019a, b).

Moreover, the cause of donor death (e.g., infection or sepsis, respiratory disease, multi-organ failure, cancer) can increase the risk of bacterial and fungal contamination (Armitage et al. 2014; Kramp et al. 2020; Röck et al. 2016), with contamination rates ranging from 2.5 to 6.4% during organ culture (Fontana et al. 2007; Ling et al. 2019). Nonetheless, the risk of keratitis or endophthalmitis developing in patients transplanted with a contaminated donor cornea is low (1.3%) (Kiatos et al. 2017).

During organ culture storage of up to 4 weeks, routine microbiological screening is performed by standard culture tests on media or via automatic systems that alert to the detection of microbial growth. We currently sample the preservation medium (*Storage*) after 6 days of organ culture and then again, following evaluation of suitability for transplantation, at 1 day after the transfer of the cornea into the transport solution (*Deswelling-Transport*) prior to delivery. The *Deswelling-Transport* solution is tested once more after processing of the cornea for endothelial or anterior lamellar keratoplasty. For example, between 2015 and 2017 we found *Candida* spp. (75%), Gram-positive and Gram-negative bacteria (20%), and unspecified yeasts and moulds (5%) in 65 isolates from positive *Storage* solutions at the end of organ culture. If the *Storage* solution results positive for microbial contamination at the first sampling period after 6 days, the cornea is discarded. Subsequent tests are performed more or less concomitantly but results may not be ready when the corneal tissue has to be sent to the surgical centre. The time window between full test result availability and delivery of tissue for transplantation is critical, with the risk of distributing unsafe corneas.

Gram stain is a simple, rapid, and low-cost method to detect bacteria, yeasts (*Candida* and *Cryptococcus* species), and moulds (*Aspergillus* species) in biological samples; it is employed to reveal slow-growing microbes or those difficult to culture and to differentiate these species from eukaryotic cells of human origin (Popescu and Doyle 1996). The Gram stain does not depend on the chemical properties of the cell wall but rather on its thickness, which explains why the thicker cells of fungi show crystal violet positive staining despite the chemical composition being different from the cell wall of Gram-positive bacteria (Murray et al. 2012).

The aim of the present study was twofold: to determine the occurrence of contaminated preservation solutions after organ culture in which amphotericin B was added to the solution (*Cold*) for hypothermic maintenance of corneas (from the retrieval site to the eye bank) before starting organ culture, and to determine the feasibility of Gram stain to detect residual microorganisms in the *Storage* solution at the end of organ culture.

## Methods

### Preservation and processing of donor corneas

**Fig. 1:**
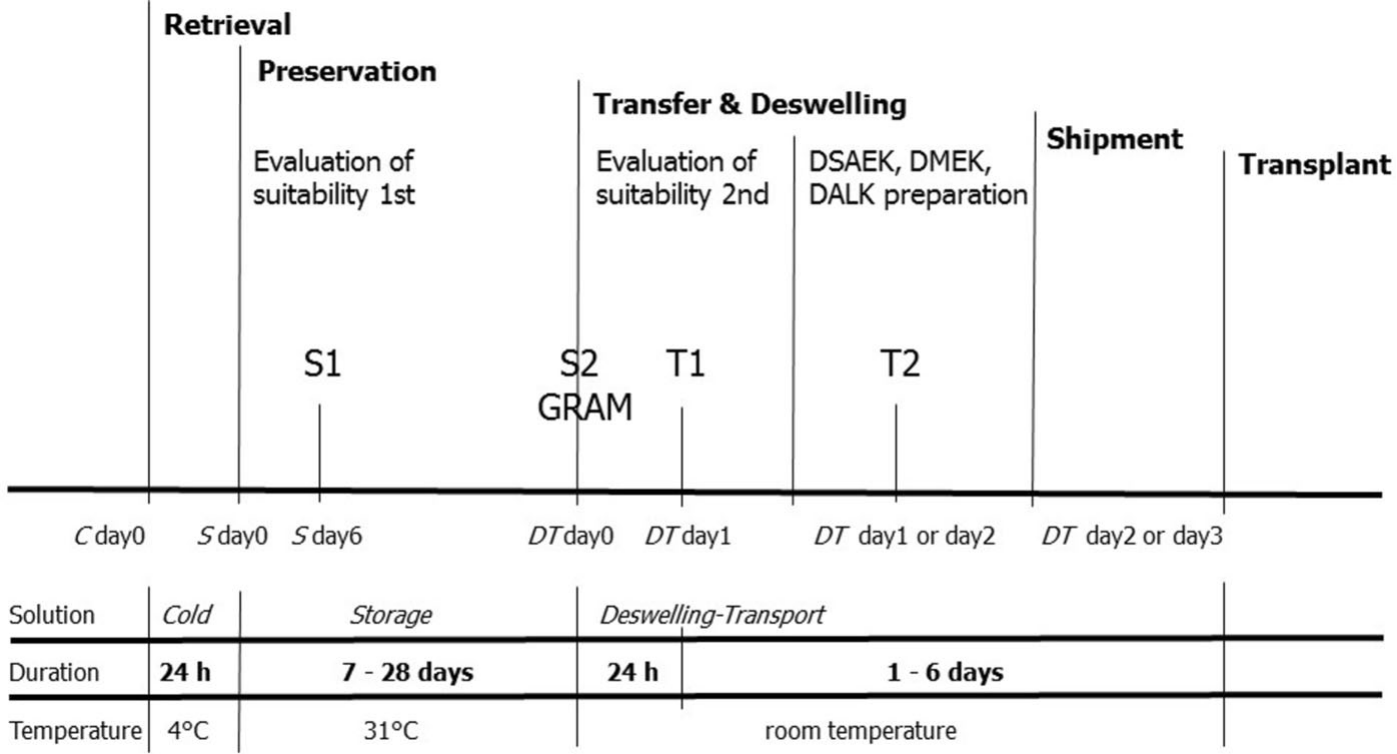
Phases of the eye bank activities and sterility testing. C denotes Cold; DT, *Deswelling-Transport*; GRAM, staining with Gram’s Method; S, *Storage*; S1, first sample during preservation in *Storage*; S2, second samples at the end of preservation and transfer of the cornea in *Deswelling-Transport*; T1, first sample after 1 day in *Deswelling-Transport*; T2, second sample of *Deswelling-Transport* after processing the cornea for lamellar keratoplasty

Figure 1 illustrates the process for organ culture of donor corneas at our institution. *Cold*, *Storage,* and *Deswelling-Transport* solutions contain MEM-Earle with HEPES 25 mM, sodium bicarbonate 26 mM, pyruvate 1 mM, glutamine 2 mM, newborn calf serum 2% v/v, penicillin G 60 µg/mL, and streptomycin 100 µg/mL. Amphotericin B 0.25 µg/mL is currently added to the *Storage* and the *Deswelling-Transport* solution. It has proven efficacy against *C. albicans* in hypothermic conditions at 2–8 °C (Tran et al. 2020). Beginning in February 2020, we supplemented amphotericin B 2.5 µg/mL to the *Cold* solution for the short-term hypothermic maintenance of donor corneas before starting preservation in organ culture. *Deswelling-Transport* contains dextran-T500 6% w/v and the cornea is maintained for at least 24 h in this solution to recover transparency and thickness before processing or grafting.

For planned surgeries, we remove corneas from *Storage* between day 14 and 35 of culture, perform light microscopy and endothelial staining, and transfer those corneas suitable for transplantation into *Deswelling-Transport*. Corneas for anterior lamellar keratoplasty (ALK) and endothelial keratoplasty (EK) are pre-cut by microkeratome, with the anterior and the posterior portion realigned in their original position for distribution, or Descemet’s membrane is manually stripped to separate the ultrathin cornea portion comprising the endothelium for Descemet membrane endothelial keratoplasty (DMEK) and then repositioned on the corneal stroma.

The corneas are placed back in their original *Deswelling-Transport* bottle and transported at room temperature in a polystyrene box. Keratoplasty is usually performed within 4 days, with a total maximum exposure to *Deswelling-Transport* of 7 days.

### Sterility testing

We test the sterility of *Storage* and *Deswelling-Transport* solutions with two validated automated systems to screen for microbial growth: Bactec 9240 (Becton–Dickinson, Franklin Lakes, NJ, USA) and HB&L (Alifax, Padua, IT) have a recommended incubation period of 7 days and 24 h, respectively (Camposampiero et al. 2013; Thuret et al. 2002). Samples are prepared under sterile conditions by injecting 3 mL of the preservation and the transport solution in the aerobic Bactec Peds Plus bottle and 3 mL in the anaerobic Bactec Plus bottle, and 0.5 mL in the HB&L culture kit for growing aerobic microorganisms. The Bactec bottles can also grow fungi (Thuret et al. 2005). We use Bactec to test the *Storage* twice: after 6 days of culture (S1) and then again after assessing cornea suitability for transplantation ≥ 7 days after S1 (S2), 1 day after transfer of the cornea into *Deswelling-Transport* (T1), and following processing of the cornea for lamellar keratoplasty (T2). We combine HB&L for aerobic microorganisms and Bactec analysis in the second *Storage* sampling (S2), whereas we use both systems only at S1, T1, and T2 tests of corneas from donors at risk of microbial contamination (Fig. 1).

### Gram stain

We performed Gram stain to test the *Storage* at the end of organ-culture preservation (S2). After transfer of the cornea into the *Deswelling-Transport* solution, a volume of 50 mL of *Storage* was centrifuged (4500 rmp, 3893×*g*) for 10 min in a sterile vial and the pellet suspended in 3 mL of sterile saline solution. A few drops of the suspended solution (mean 200 µL) were distributed over a microscope slide and air-dried for about 30 min. The sample was heat-fixed by passing the slide over a flame until the underside was warm to the touch; the slide was then inserted into an automatic slide stainer (Wescor Aerospray Gram 7321, Delcon, Milan, IT) for automatic staining.

The microorganisms were stained with crystal violet (Gram-positive), which was then fixed to the wall with iodine, while the dye on the back of the slides was wiped clean with a cloth soaked in alcohol. To conclude the procedure, safranin was used to stain the decolorized Gram-negative microorganisms. Gram-positive microorganisms appear purple or blue and Gram negatives pink or red. The slides were observed under a light microscope (magnification 400× and 1000×); samples were defined positive if they showed bacterial or fungal microorganisms differing by colour and cell morphology from eukaryotic cells of corneal origin.

### Statistical analysis

We recorded the incidence of positive cultural microbial tests performed on *Storage* and *Deswelling-Transport* solutions after completion of organ culture and compared the results for the year 2020 with the historical data (2015–2019), as well as in corneas from at-risk donors compared to corneas from not-at-risk donors in the year 2020. Incidence is expressed as number per 100 corneas.

The χ2 test was used to evaluate differences between year 2020 and the historical data (2015–2019) and between at-risk and not-at-risk donor corneas. The significance level was set at 0.05 and 95%. Confidence intervals were calculated Statistical analysis was performed using STATA statistical software version 13 (StataCorp LP, College Station, TX, USA).

## Results

**Fig. 2:**
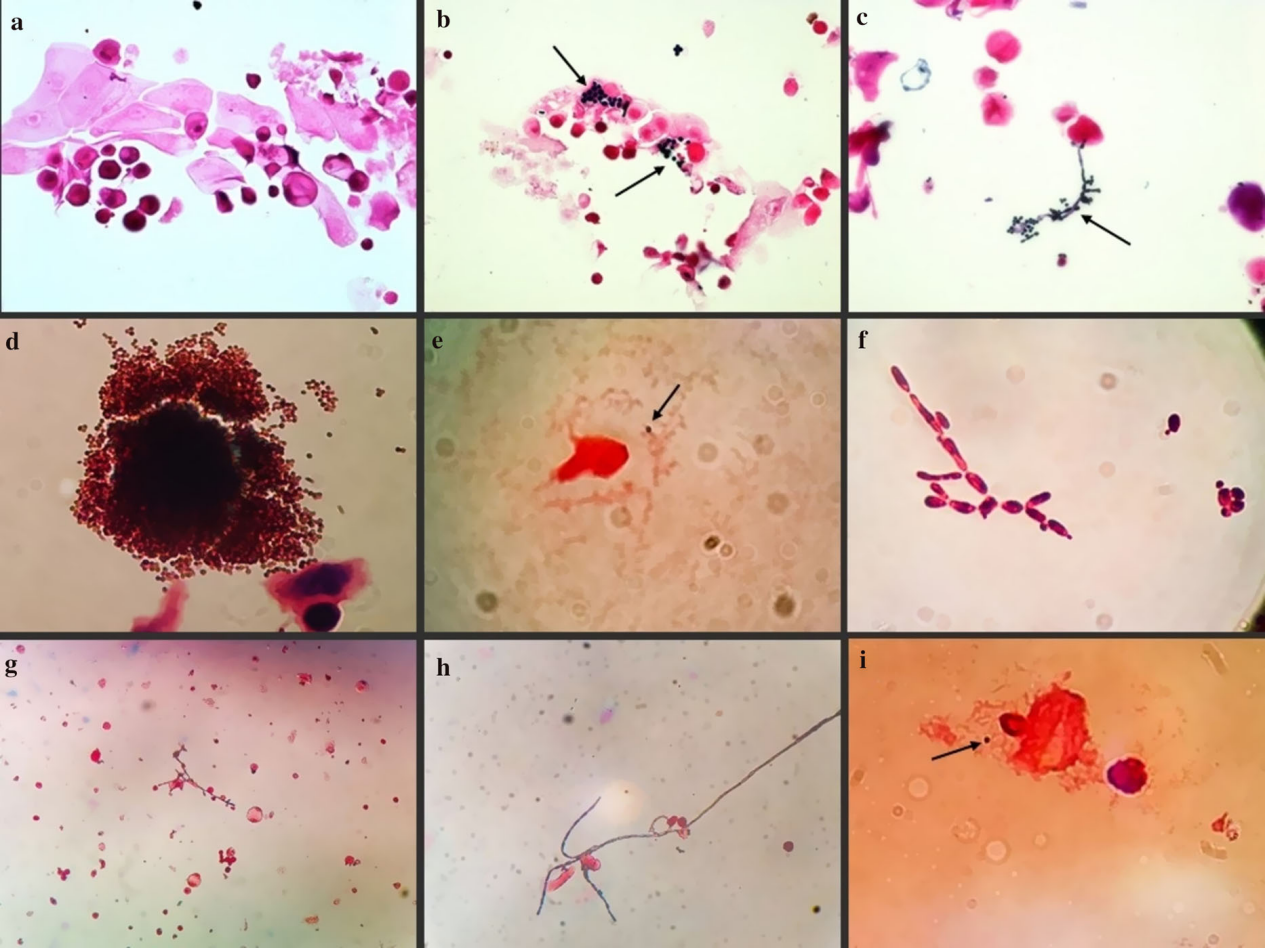
Example images of Gram stain results. **a** Superficial squamous corneal epithelia and cells with vacuolated cytoplasm of likely conjunctival origin; Gram staining negative for microorganisms; **b** aggregates of sporiform Gram-positive elements, likely *Candida* spp.; **c** pseudohyphae and blastospores of a Gram-positive yeast, likely *Candida* spp.; **d** massive aggregates of blastospores; **e** Gram-positive cocci and squamous epithelial corneal cells; **f** pseudohyphae and chlamydospore of *Candida* spp.; **g** fungal hypha among epithelial corneal cells; **h** enlarged detail of fungal hypha; **i** enlarged image of Gram-positive cocci. Images **a**–**c**, magnification ×400; images **d**–**g**, magnification ×1000; images **d**–**i** acquired directly from the eyepiece of the light microscope

Table 1 presents the results of culture tests for the 6-year period 2015–2020. We found no substantial difference in the number of donor corneas discarded due to a positive test result at S1 in 2020 (N = 171, 3.9%) compared to the mean of 155 (3.5%) for the previous five years (*p* = 0.21). The number of positive *Storage* and *Deswelling-Transport* solutions after culture tests of suitable corneas was 15 (0.5%) in 2020 compared to a mean of 37 (1.2%) positive solutions in the period 2015–2019 (*p* < 0.01). The difference was likely due to supplementation of the *Cold* solution with amphotericin B. There were 10 (1.0%) positive samples in the cohort of 998 (32.8%) donor corneas at risk and 5 (0.2%) in the 2046 (67.2%) corneas not at risk (*p* < 0.01). All at-risk corneas tested positive also at Gram stain and the test results were available 1 to 3 days before those from the culture tests performed according to our current protocol (Table 2). We identified by morphology Gram-positive cocci in 6 samples, fungal spores in 3, and *Candida* spp. in one sample (Fig. 2).

**Table 1:** Results of sterility tests and postoperative adverse reactions recorded in 2015–2020 period

Year	Stored corneas	S1 +	Suitable corneas	S2 + only	T + only	S2 + and T +	Positive corneas total	Positive corneas shipped	Positive corneas grafted	AR^§^
	N	N (%)	N	N (%)*	N (%)*	N (%)*	N (%)*	N (%)**	N	N
2015	3822	181 (4.7)	2674	13 (0.5)	16 (0.6)	6 (0.2)	35 (1.3)	15 (42.9)	1	1
2016	4368	156 (3.6)	2862	12 (0.4)	8 (0.3)	4 (0.1)	24 (0.8)	5 (20.8)	0	0
2017	4606	110 (2.4)	3125	31 (1.0)	9 (0.3)	3 (0.1)	43 (1.4)	7 (16.7)	5	1
2018	4557	143 (3.1)	3022	22 (0.7)	10 (0.3)	9 (0.3)	41 (1.4)	11 (26.8)	2	0
2019	4903	184 (3.7)	3154	21 (0.7)	13 (0.4)	8 (0.2)	42 (1.3)	11 (26.2)	1	1
2020	4414	171 (3.9)	3044	8 (0.3)	2 (0.1)	5 (0.2)	15 (0.5)	2 (0.1)	0	0
**Total**	**26,670**	**945 (3.5)**	**17,881**	**107 (0.6)**	**58 (0.3)**	**35 (0.2)**	**200 (1.1)**	**51 (25.5)**	**9**	**3**

Bold represent the total values in each column

*Percentage on suitable corneas; **Percentage on positive corneas total; AR, adverse reaction; ^§^Of the 3 cases of endophthalmitis post keratoplasty, that in 2015 was associated to *Fusarium* spp. contamination, that in 2019 to *Candida* spp., and the one in 2017 was of undetermined aetiology though reportedly due to the donor cornea according to the surgeon opinion, despite negative microbial testing at the eye bank

**Table 2:** Characteristics of microbial-positive corneas in 2020

No.	Intended graft	Gram stain	S2 Bactec	S2 HB&L	T1 Bactec	T1 HB&L	Results of Gram before Bactec/HB&L (days)	Shipped
1	PK	Cocci Gram+	+	+	ND	ND	1	No
2	PK	*Candida* spp	+	+	−	ND	1	No
3	PK	Fungal spores	+	−	+	ND	3	No
4	PK	Cocci Gram+	+	−	+	−	2	No
5	DSAEK	Fungal spores	+	−	−	ND	2	No
6	PK	Fungal spores	+	+	ND	ND	1	No
7	PK	Cocci Gram +	+	+	ND	ND	1	No
8	PK	Cocci Gram+	+	+	ND	ND	1	No
9	PK	Cocci Gram+	+	−	ND	ND	1	No
10	PK	Cocci Gram+	+	−	+	ND	3	No
11	PK	NA	+	+	ND	ND	NA	No
12*	DSAEK	NA	−	−	+	−	NA	Yes**
13*	DSAEK	NA	−	−	+	ND	NA	Yes**
14	PK	NA	+	−	+	ND	NA	No
15	PK	NA	+	+	+	ND	NA	No

*Cultural tests positive also at T2; **Not transplanted; DSAEK denotes Descemet stripping automated endothelial keratoplasty; *NA* not applicable, *ND* not done, *PK* penetrating keratoplasty

*Deswelling-Transport* at T2 was evaluated only for two corneas (nos. 12 and 13) processed for endothelial keratoplasty in corneas from not-at-risk donors. Both corneas were distributed for surgery while the T1 and the T2 sterility tests were ongoing; however, grafting was not performed because the test results became available (positive T1 and T2 in both cases) before the corneas arrived at the surgical centre. Additional Gram stain of the *Deswelling-Transport* of these two corneas showed Gram-positive cocci of *Streptococcus* spp.

## Discussion

In the present study, we compared two methods potentially useful for improving the microbial safety of human corneas for transplantation. We discarded because of positive microbial tests at the end of organ culture less than half of the mean number of corneas discarded annually in the period 2015–2019 and we observed that amphotericin B 2.5 µg/mL added to the *Cold* solution is effective in reducing the primary contamination load. Furthermore, we compared the effectiveness of Gram stain and standard culture tests for early identification of residual microbial contaminants in the *Storage* solution. We noted that Gram stain yielded positive results 1 to 3 days before the culture tests.

In general, antibiotic activity against fungi in preservation solutions is not seen at the first sterility test (S1) after the start of organ culture because of the slow growth of microbes and the latency in detection by culture systems. The effect of amphotericin B on the initial microbial load became evident in the test at the end of organ culture (S2) since prolonged storage at 30–37 °C increases the likelihood of microbial growth and the proliferation of microorganisms, mainly molds and yeasts. Direct microscopy detection of fungal structures following Gram stain improved the real-time identification of slow-growing microbes in 4 out of 10 positive stains in our study.

Contamination remains a problem, however, as shown by the two cases of microbial positive corneas pre-cut for DSAEK that did not undergo Gram stain. Gram stain should be extended to low-risk corneas to assess the accuracy of this procedure in comparison to standard culture methods in the early detection of microbial contaminants and hence prompt discarding of the corneas.

The growing demand for human corneas for transplantation and the expanding involvement of eye banks in the supply of pre-prepared ready-to-use corneal tissues for endothelial or anterior lamellar grafts, place added time pressure on the eye bank, chiefly during the deswelling and the delivery stage. The need to transplant tissues within 4 days after processing for lamellar keratoplasty limits the efficacy of culture sterility tests because the corneas are delivered before the culture tests are concluded at S2 and T1/T2. Microbial culture tests based on growth-dependent systems can create overly long waiting times. So how can we ensure both safety and prompt delivery?

Our study results could open a new scenario in the evaluation of the sterility of organ-cultured corneas: coupling the Gram stain procedure with culture systems could align organ culture with organ delivery. The pressing demand for corneas and the ongoing shortage of donor corneas have shortened the preservation time (in 2020 the average in our institution was 14 days); less time is allotted for deswelling and transport of corneas for EK and DSAEK. In corneas processed for ALK or EK/DMEK, sterility at T1 and T2 may coincide or differ by a few hours, with delivery of the graft—to ensure its quality—on the same or the next day. The non-reactivity of the *Storage* solution at Gram stain and the maintenance of asepsis during processing for ready-to-use corneas could ensure a sufficient level of microbial safety and make the current culture tests on the *Deswelling-Transport* redundant.

When we process corneas from *Storage* to *Deswelling-Transport* (sterility testing is mandatory when transferring the cornea) and prepare them for ALK or EK/DMEK, we sample at T1 and T2 so that we can be sure that fungal contaminants have been eliminated and the corneas from Gram-positive *Storage* have been discarded, although positive results can be expected.

Finally, given the worldwide shortage of cornea donations, Gram stain should be adopted for rapid identification of microbial contaminants. This would aid surgeons in selecting appropriate preventive antibiotics and antifungal drugs, though grafts with a positive rim culture do not tend to result in postoperative infections (Li et al. 2019a, b).

A major limitation of Gram stain is that it is operator-dependent; nevertheless, we believe that it can be efficiently managed for consistent and reproducible results by automatic staining of slides and that basic microbiology and histology skills can help shorten the learning curve for microbial identification.

Based on our data, supplementation with amphotericin B 2.5 µg/mL in the short-term hypothermic maintenance solution and Gram staining of storage solutions at the end of organ culture offer successful strategies to reduce the initial microbial load, to detect residual microbial contaminants promptly, and to discard contaminated corneas. Both strategies may help to improve the safety of corneal tissues and reduce the rate of postoperative infectious keratitis caused by slow-growing microorganisms such as moulds and yeasts.

## Data Availability

Data and material are available for users granted access.
